# A qualitative study on compassion fatigue experience and influencing factors of geriatric nurses

**DOI:** 10.12669/pjms.41.4.9051

**Published:** 2025-04

**Authors:** Yang Dong, Yuee Huang, Danli Li, Xiaoling Li, Hongying Rao

**Affiliations:** 1 Yang Dong, MSN, Geriatrics Department, Guangzhou First People’s Hospital, No.1 Panfu Road, Yuexiu District, Guangzhou 510000, China; 2 Yuee Huang, BS Nursing, Geriatrics Department, Guangzhou First People’s Hospital, No.1 Panfu Road, Yuexiu District, Guangzhou 510000, China; 3 Danli Li, BS Nursing, Geriatrics Department, Guangzhou First People’s Hospital, No.1 Panfu Road, Yuexiu District, Guangzhou 510000, China; 4 Xiaoling Li, MSN, West China School of Nursing, Sichuan University, No. 37, Guoxue Lane, Wuhou District, Chengdu 610000, China; 5 Hongying Rao, BS Nursing, Geriatrics Department, Guangzhou First People’s Hospital, No.1 Panfu Road, Yuexiu District, Guangzhou 510000, China

**Keywords:** Compassion Fatigue, Geriatrics, Nurses, Qualitative Study

## Abstract

**Objective:**

With the rapid development of an aging society, the workload of geriatric nurses is increasing, but their psychological health issues are not receiving enough attention. This study explores the experience of compassion fatigue and its influencing factors among geriatric nurses, and provides targeted recommendations.

**Methods:**

This qualitative study, conducted at the Guangzhou First People’s Hospital. Sixteen nurses of a geriatric department were selected using purposive sampling and subjected to semi-structured in-depth interviews. The study was carried out from September 2022 to February 2023, Colaizzi’s method was used to analyze the qualitative data obtained from the interviews.

**Results:**

Three main themes were extracted from the analysis: compassion fatigue greatly affects my personal mentality, various things can cause nurses to experience compassion fatigue, and compassion fatigue causes many problems in the lives and work of nurses, including ten sub-themes.

**Conclusion:**

This qualitative study reports nurses in geriatric department are prone to compassion fatigue. The factors influencing nurses’ compassion fatigue need to be correctly identified so that targeted pre-control strategies can be adopted to promote nurses’ physical and mental health, increase compassion satisfaction and reduce burnout.

## INTRODUCTION

Empathy fatigue and compassion fatigue are becoming common among healthcare professions. However, there are some differences between empathy and compassion. Empathy fatigue refers to the state in which an individual feels exhausted emotionally and physically due to overly identifying with the feelings of others. It involves the internalization of others’ emotions by the individual, which may lead to the individual feeling emotionally disconnected or numb.^1^ Compassion fatigue is the emotional, physical and mental exhaustion that occurs as a result of long-term care for others who are suffering. It involves the continuous attention and response to the pain of others, which may cause the individual to be indifferent to the suffering of another individual.^2^

Compassion constitutes the fundamental and intrinsic essence of nursing practice. Clinical nurses, being in direct and extensive contact with patients, are consistently exposed to a multitude of traumatic events over prolonged periods.^3^ When the cumulative level of compassion surpasses their capacity for coping and recuperation, it leads to compassion fatigue, which can elicit a range of physiological, social, emotional, mental, and cognitive repercussions on nurses.^4^

China’s society is experiencing rapid aging, leading to increasing concerns regarding the health issues faced by the elderly.^5^ Geriatric nurses, who play a pivotal role in providing care for elderly patients in hospitals, face higher demands for compassion. Therefore, it is crucial to prevent compassion fatigue, as it can potentially compromise the quality of care for elderly patients.^6^ Therefore, there is a need to explore and understand compassion fatigue among geriatric nurses as it serves as a crucial reference for the development of intervention strategies to effectively address compassion fatigue. This, in turn, improves quality of care for elderly patients and geriatric nurses’ quality of life.

## METHODS

This qualitative study was conducted at Guangzhou First People’s Hospital from September 2022 to February 2023. The reporting of this study complied with the consolidated criteria for reporting qualitative research (SRQR) recommendations.^7^

### Participants:

To ensure maximum diversity among the interviewees, geriatric nurses with different working years, positions, and job titles were selected, purposive sampling technique was used. This study subjects were 16 geriatric nurses (coded N1-N16), all interviewees’general information is shown in the Table-I.

**Table-I:** Sociodemographic characteristics of the participants (n).

	FGs (n = 16)
*Gender*	
Women	14
Man	2
*Age(years)*	
<30	5
31-50	9
>51	2
*Employment status*	
Temporary or long-term	5
Statutory or permanent	11
*Work experience(years)*	
<10	6
11-20	7
>21	3

FG: focus group.

### Ethical approval:

This study has been approved by the ethics committee of Guangzhou First People’s Hospital (K-2023-032-01, Date of approval: March 16, 2023).

### Inclusion criteria:

- Must be a practicing nurse with actual patient care experience.
- Has at least one year of nursing work experience.
- Has willingness to participate in this study.

### Exclusion criteria:

- No actual patient care experience.
- No compassion and inability to understand and experience the emotional state of others.
- Diagnosis of mental or other cognitive and emotional disorders.
- Nurses who did not complete the interview for various reasons.

### Data collection:

Each interview lasted between 20-40 minutes, and ended when no new information was generated. The interview outline consisted of the following questions:

- What experiences of compassion fatigue have you had while continuously investing emotions in your nursing work?
- What factors have influenced the occurrence and exacerbation of the aforementioned compassion fatigue experiences?
- What impact have these compassion fatigue experiences had on you after continued development?
- How do you handle various emotional disturbances when working?

To ensure the precision of data collection in the interviews, several techniques were implemented:

- Active Listening: Researchers actively engaged in attentive listening to comprehensively comprehend participants’ experiences.
- Probing Questions: Additional questions were employed to delve deeper into participants’ responses and clarify any ambiguities.
- Audio Recording and Transcripts: Interviews were recorded with participants’ consent and transcribed verbatim to guarantee accuracy.
- Field Notes: Detailed field notes were taken by researchers during and after interviews to capture non-verbal cues and contextual information.
- Data Verification: Transcripts underwent participant review for validation, ensuring the accuracy of data representation.

### Data analysis:

Following each interview, the interview recordings were transcribed verbatim within 24 hours and the transcribed texts were imported into Nvivo 12.0 software, while general demographic information of the interviewees was recorded in Excel. For data analysis, the Colaizzi method was employed to systematically code, classify, synthesize, and refine the interview content.^8^

## RESULTS

The main themes, eight sub-themes, and units of meaning that emerged from the discourses are described in Table-II.

**Table-II:** Themes, sub-themes, and units of meaning derived from data analysis.

Compassion fatigue had a great impact on personal mentality	Sadness	The care of elderly patients and grief of their families can cause sadness for nurses, impacting their mental state and taking time to recover.
	Numbness and indifference	Nurses in geriatric ward face the challenge of empathetic exhaustion and need to spend time to recover from the stress of nursing, also the understanding of patient’s family members towards their work is crucial.
Various factors contribute to nurse compassion fatigue	Characteristics of elderly patients	Elderly patients with persistent physical symptoms due to their advanced age caused nurses to ignore their suffering. The critical condition of elderly patients led to complex nursing tasks.
	Personal and family factors	Personal and family factors affected compassion-related emotional experiences of junior nurses facing critically ill elderly patients, including sadness, depression, crying, anxiety, nervousness, nightmares, flashbacks, avoidance, numbness, etc.
	Working environment	Nurses suffered from burnout due to heavy workloads and inadequate rest, and workplace violence exacerbates the psychological, physical, and social burdens on nurses.
Compassion fatigue brings many difficulties to the lives and work of nurses	Impacts on personal health	Compassion fatigue can harm the physical health of geriatric nurses, and took time for self-care to aid in healing.
	Interferes with family harmony	Nurses’ emotional exhaustion can negatively impact their family relationships and reduce their quality of life, affecting not only themselves but also their loved ones.
	Impacts on work	Compassion fatigue affects the emotional investment of geriatric nurses in their work, leading to low work enthusiasm, poor quality of nursing, and intention to resign.

### Theme-1: Compassion fatigue had a great impact on personal mentality.

### Sadness:

N1: “ When you saw the patient getting worse day by day, it’s just tough to stay positive.” N3: “ We had been providing care for the patient since their admission to the hospital, so if they were to suddenly pass away, it might take us a day or two to recover from our sadness.”

### Numbness and indifference:

N13: “ The passion we had when we first started working has been worn down by day-to-day work. We feel that our work is quite mechanical now.”

### Theme-2: Various factors contributed to nurse compassion fatigue.

### Characteristics of elderly patients:

N6: “ Patients with persistent physical symptoms due to their advanced age caused me to ignore their suffering.” N5: “Most elderly patients cannot communicate effectively with me due to illness, which leaded me to ignore their feelings in daily nursing.” N8: “ The complex condition of elderly patients often brought more nursing tasks, so that I had no time to pay attention to the feelings of patients.”

### Personal and family factors:

N11: “I am a new nurse with limited abilities, and I felt more powerless than compassion when facing elderly patients with deteriorating conditions.” N7: “ I can adjust my emotions quickly. I may feel sad at the time, but it won’t last long. This is related to my personality.” N10: “If something is going on with my family during this time, my mood will not be good, and I won’t be able to invest as much in the patients.”

### Working environment:

N13: “All kinds of things are endless, especially when the entire room was filled with critically ill patients. I really wanted to quit.” N4: “ The night shift is too long, from 20:00 to 8:00 the next day. The whole diet and sleep schedule are all messed up. It’s too tiring.” N2: “Sometimes patients used the doctor’s words to contradict me, even though what the doctor said was not correct and interferes with my work, which leaded to displeasure between me and the doctor.” N12: “ Despite the fact that there is still a chance for critical patients to recover, I sometimes have conflicts with doctors when they adopt a passive treatment plan, as I feel that all my previous efforts have been in vain.”

### Theme 3: Compassion fatigue brought many difficulties to the lives and work of nurses.

### Impacts on personal health:

N16: “ This negative emotion affected my rest and physical health. So after work, I must put work affairs aside and did something I enjoyed.”

### Interferes with family harmony:

N14: “ Sometimes when I went home and talked calmly with my family about the death of a patient I had cared for a long time, my children thought I was cold-blooded.” N5: “With the increase of working years, I became more rational. However, my family thought that I lack compassion and had become somewhat numb. It affected my relationship with them to some extent.”

### Impacts on work:

N15: “I was joking with another patient in the same ward when the family of a critical patient I was in charge of came to visit. They complained that I was very cold-hearted, even though the patient’s condition was stable at the moment.” N9: “ Due to my workload, some patients may perceive me as indifferent. It may hinder the effectiveness of my nursing practice.”

## DISCUSSION

This study found that longer years of work experience is linked to higher compassion fatigue. Compassion fatigue, common among geriatric nurses, leads to emotional exhaustion, occupational burnout, and reduced personal accomplishment. These findings align with international research, showing the widespread challenge of compassion fatigue among healthcare professionals, especially during the COVID-19 pandemic, which has profoundly affected their personal and professional lives. This study also noted that male nurses reported higher levels of compassion fatigue. And workplace violence may exacerbate compassion fatigue.

The findings of this study align with international researches, indicated the pervasive nature of compassion fatigue among healthcare professionals worldwide.^9^,^10^ Particularly during the COVID-19 pandemic, compassion fatigue had exerted a profound impact on both personal and professional aspects of healthcare providers’ lives, amplified the susceptibility to compassion fatigue and potentially compromising the quality of patient care.^11^

Several studies have indicated a negative correlation between years of work experience and the occurrence of compassion fatigue.^12^-^14^ However, findings from this study’s interviews suggested that despite their enhanced ability to cope with occupational stress, senior nurses may still encounter emotional exhaustion, this might be associated with the complex circumstances and higher care demands of elderly patients. In another study, no association was found between compassion fatigue and years of experience.^15^ This disparity might be attributed to the variations in the professional background, working environment, and cultural factors of nurses in different studies.

In this study, it was observed that male nurses exhibited a higher degree of compassion fatigue during the interview process, potentially attributed to societal biases, professional status disparities, and salary discrepancies. This finding aligns with prior research indicating that male nurses experience elevated levels of compassion fatigue compared to their female counterparts.^16^

Workplace violence can significantly contribute to the development of compassion fatigue among nurses.^17^ A study indicated that 71.9% of healthcare professionals have experienced workplace violence, with verbal assaults and threats being the most prevalent forms.^18^ And another study revealed that one of reasons for nurses experiencing compassion fatigue is the interpersonal frictions with members of medical team.^19^ Similarly, in our interviews, our group found that most elderly nurses had experienced verbal conflicts with doctors due to disagreements on treatment decision-making.

Most nurses lack awareness of compassion fatigue and show little concern for issues related to psychological health.^20^ And this study revealed that geriatric nurses typically lack effective strategies to address compassion fatigue, frequently permitting its progression and resorting to methods of distraction or passive avoidance. Compassion fatigue significantly impacts nurses’ physical, psychological, and social adaptation. Recurring physical and mental conditions directly impair the quality of nurses’ work and life, leading to weakened professional identity, disengagement from work, self-focused behavior, increased interpersonal conflicts, and ultimately a desire to leave the nursing profession.^21^

Based on the interview content and research results of this study, our research group recommends that hospitals allocate additional resources, including training, counseling, and support groups, to assist nurses in managing compassion fatigue. Furthermore, hospitals should evaluate and potentially reduce nurses’ workloads while enhancing stress management techniques and tools for medical staff in the geriatric department. Lastly, it is crucial to encourage nurses to prioritize self-care and seek support when necessary to prevent compassion fatigue and enhance their overall well-being.

The finding of this study provides insights into the unique experiences and challenges faced by geriatric nurses, which can differ from those in other healthcare settings. Furthermore, in this qualitative study, face-to-face interviews were conducted, enabling the observation of nonverbal cues such as facial expressions, eye contact, and body language, which consists a substantial strength of this study.

### Limitations:

This study provides valuable insights into the experiences of empathy fatigue among geriatric nurses and its influencing factors, but also has some limitations. Firstly, due to the small sample size, which only included 16 nurses, the study’s findings are limited in universality and may not fully represent the experiences of all geriatric nurses. Secondly, the study was conducted in a single medical facility, Guangzhou First People’s Hospital, which may affect the study’s broad applicability, and the experiences of nurses in different regions and different levels of medical institutions may be different. Finally, the study was completed within six months, long-term research topic experience will be a worthwhile path to pursue in the future.

## CONCLUSION

The findings of this study make a significant contribution to the nursing field’s comprehension of the intricate nature of compassion fatigue experience and its influencing factors among geriatric nurses. It is anticipated that these findings will inform the development of targeted interventions aimed at addressing these challenges and enhancing the quality of care provided to older adults.

### Authors’ Contribution:

**YD:** Literature search, Drafting of the manuscript and obtained funding.

**XL, HR and YD:** Study concept and design, critical revision of the manuscript for important intellectual content and study supervision

**YD and DL:** Acquisition of data. Critical review.

**YD, DL, YH, XL and HR:** Literature search, Analysis and interpretation of the data.

**XL, HR and YH:** Administrative, technical, or material support. Critical analysis.

All authors have approved the final version and are accountable for the integrity of the study.
